# Combination of 5‐Fluorouracil and Thymoquinone for Enhanced Cytotoxicity, Genotoxicity and Apoptosis In Colorectal Cancer: In Vitro and In Vivo Studies

**DOI:** 10.1002/jbt.70276

**Published:** 2025-04-30

**Authors:** Eray Metin Guler, Kubra Bozali, Onder Huseyinbas, Mert Celikten, Abdurrahim Kocyigit

**Affiliations:** ^1^ Department of Medical Biochemistry, Haydarpasa Numune Health Application and Research Center İstanbul Türkiye; ^2^ Department of Medical Biochemistry, Faculty of Hamidiye Medicine University of Health Sciences Turkey Istanbul Türkiye; ^3^ Department of Medical Biochemistry Bezmialem Vakif University, Faculty of Medicine Istanbul Türkiye; ^4^ Department of Medical Biochemistry University of Health Sciences Turkey, Hamidiye Institute of Health Sciences Istanbul Türkiye; ^5^ Experimental Application and Research Center Bezmialem Vakif University Istanbul Türkiye

**Keywords:** 5‐Fluorouracil, colorectal cancer, In Vivo, IVIS, thymoquinone

## Abstract

Research on the effects of herbal‐derived natural active substances on cancer treatment and their combination with conventional treatments has intensified. This study analyzed the cytotoxic, genotoxic, apoptotic, and anticancer effects of combined treatment with 5‐Fluorouracil (5‐FU) and thymoquinone (TQ) on colorectal cancer. Cytotoxicity was evaluated using the ATP assay, DNA damage was assessed through the comet assay, apoptosis was measured via acridine orange/ethidium bromide staining and annexin V‐FITC dye, and the expression of proapoptotic and antiapoptotic proteins was determined by western blot analysis. Transfected LoVo cells were injected subcutaneously into nude mice, and following treatment, oxidative stress and inflammation markers were examined in blood samples, while growth factors and vascularization markers were analyzed in tissue samples. The combination therapy at low concentrations resulted in increased cytotoxicity, DNA damage, apoptosis, and intracellular reactive oxygen species (*p* < 0.001), while simultaneously decreasing mitochondrial membrane potential and glutathione levels (*p* < 0.001), in comparison to monotherapy with TQ or 5‐FU. Additionally, tissue levels of TGF‐β1 and VEGF‐α were significantly reduced (*p* < 0.001). Results demonstrates that while TQ or 5‐FU alone have notable anticancer effects, their combination offers greater efficacy in mitigating molecular changes in both In Vitro and In Vivo models. Future studies should focus on optimizing the formulation, understanding the molecular mechanisms, and evaluating the efficacy and safety of the TQ and 5‐FU combination across different cancer types.

## Graphical abstract

The study shows that combination treatment with thymoquinone and 5‐Fluorouracil significantly inhibits tumor growth in a colon cancer xenograft model as shown by bioluminescence imaging. This suggests a potential therapeutic synergy between thymoquinone and 5‐Fluorouracil to enhance anticancer efficacy (This graphical abstract was created by BioRender).

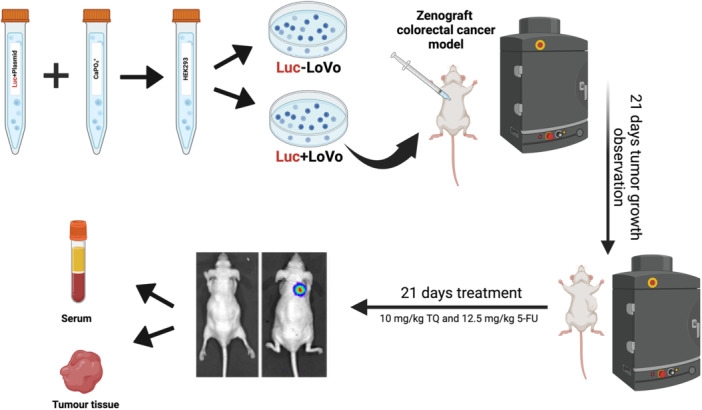

**Abbreviations**
5‐FU: 5‐Fluorouracil
CC: colorectal cancer
EDTA: Ethylene Diamine Tetra Acetic Acid
GSH: glutathione
IL‐1β: Interleukin‐1β
IL‐6: Interleukin‐6
IVIS: In Vivo Imaging System
KCl: Potassium Chloride
MCP‐1: monocyte chemoattractant protein‐1
MMP: mitochondrial membrane potential
NaCl: Sodium Chloride
NS: *Nigella sativa*
NT: native thiol
OSI: oxidative stress index
RLU: relative luminescence
ROS: reactive oxygen species
TAS: total antioxidant status
TGFβ1: transforming growth factor beta 1
TNF‐α: tumor necrosis factor‐alpha
TOS: total oxidant status
TQ: thymoquinone
TT: total thiol
VEGF‐α: vascular endothelial growth factor Alpha

## 1 Background

Colorectal cancer (CC) is the most prevalent malignancy after lung, liver, breast, and prostate cancer [1]. Annually, over one million cases of CC are diagnosed worldwide [2]. CC is one of the most common and life‐threatening malignancies globally, characterized by exceptionally high incidence and mortality rates [3, 4].

In patients with advanced‐stage CC, chemotherapy is typically administered following surgical intervention. For CC, the FOLFOX19 protocol, which includes 5‐Fluorouracil (5‐FU), leucovorin (folinic acid or LV), and oxaliplatin or the FOLFIRI protocol, which consist of 5‐FU, leucovorin, and irinotecan, are commonly preferred. In some cases, chemotherapy can be administered before surgery for patients with advanced CC who are initially inoperable due to invasive tumors; this approach is known as neoadjuvant therapy [5]. Although, many CC patients initially respond to chemotherapy, the disease often progresses following first‐line treatment. In such instances, patient may be administered an alternative chemotherapy regimen known as “second line” treatment. Early detection of disease recurrence is crucial for improving survival rates, as late diagnosis can increase the mortality rate of CC to 50%. Typically, patients typically undergo posttreatment evaluations, following the completion of chemotherapy [6, 7].

In recent years, naturally occurring compounds have become increasingly important in identifying potential candidates for drug development to combat various diseases [8]. As the global incidence of cancer rises and drug resistance becomes more prevalent, the demand for novel and effective medications continues to grow. Despite the lengthy and costly drug discovery processes, plant‐derived drugs remain a significant source for new therapeutic agents [9].

*Nigella sativa* (NS), commonly known as black seed or black cumin, is a remarkable plant from the Ranunculaceae family known for its healing properties. While NS is native in Europe, Africa, and Asia, it is particularly used as a spice and medicinal remedy in India, Pakistan, and Turkey [10]. The therapeutic benefits of NS primarily derive from its abundant active compound thymoquinone (TQ). TQ makes up approximately 25% of the volatile oil in NS and is largely responsible for its therapeutic effects [11]. Besides TQ, black cumin contains various active compounds such as thymohydroquinone, dithymoquinone, p‐cymene, carvacrol, 4‐terpineol, t‐anethole, sesquiterpene longifolene, alpha‐pinene, and thymol. TQ, also known as 2‐isopropyl‐5‐methylbenzoquinone (C_10_H_12_O_2_, molecular weight: 164.20 g/mol), is present in NS at a concentration of 30‐50% [12]. The pharmacological potential of TQ has been extensively studied, demonstrating a wide range of activities, including antibacterial, antifungal, antioxidant, antidiabetic, anti‐inflammatory, analgesic, gastrohepatic, nephroprotective, pulmonary, and testicular protective effects [13]. Among its various pharmacological activities, NS has gained particular attention for its anticancer properties, with studies indicating its potential to induce apoptosis, inhibit tumor growth, and enhance the efficacy of conventional chemotherapeutic agents. In this study, we aimed to evaluate the antitumor effects of the combined treatment with TQ, the active ingredient of black cumin, and 5‐FU, an anti‐neoplastic agent commonly used in clinical practice, on colorectal cancer. This evaluation was conducted through both In Vitro cell culture studies and In Vivo experiments using a colorectal cancer model in transgenic nude mice with xenografts derived from transfected cells.

## 2 Materials and Methods

### 2.1 Reagent and Chemicals

Sodium Chloride (NaCl), Potassium Chloride (KCl), Sodium Hydrogen Phosphate Dihydrate (Na_2_HPO_4_.2H_2_O), Potassium Dihydrogen Phosphate (KH_2_PO_4_), Ethylene Diamine Tetra Acetic Acid (EDTA), Tris HCl, Tris Base, d‐luciferin and Thymoquinone were purchased from Sigma Aldrich (St Louis, MO, USA). The chemotherapy agent 5‐FU was obtained from Deva (Istanbul, Türkiye). The primary antibodies (Bax (Cat. No #2772 T), Bcl‐2 (Cat. No #15071 T), Caspase‐3 (Cat. No #9662S), Caspase‐9 (Cat. No # 9502S), β‐actin (Cat. No #4967S) from Cell Signaling Technology, USA) and secondary antibodies (anti‐mouse IgG (Cat. No #7076P2), and anti‐rabbit IgG (Cat. No #7074), were also sourced from Cell Signaling, USA.

### 2.2 Cell Culture and Maintenance

Human LoVo (ATCC® CCL‐229) adenocarcinoma and CCD‐18Co (ATCC® CRL 1459^TM^) healthy colorectal cell lines (passage 2), both commercially available from the American Type Culture Collection (ATCC) were utilized in this study. LoVo cells were cultured in F12K medium supplemented with 10% FBS and 1% P/S, while CCD‐18Co cells were maintained in E'MEM medium supplemented with 10% FBS, 1% l‐glutamine, and 1% P/S at 37℃ in an incubator with 5% carbon dioxide and 95% air.

### 2.3 Transfection of Lovo Cells

Luc plasmids (psPAx2 # 12260 and pMD2.G Plasmid # 12559) were identified in the bacteria on agar plates. The bacteria were cultivated separately, and a single colony was selected and isolated using a plasmid isolation kit (Macherey‐Nagel M&N).

LoVo cells were seeded into 24 wells at a density of 3 × 10^4^ cells/well. After 24 h, 50 μL of the virus‐containing medium, previously aliquoted post‐transduction and stored at −80°C, was added to 950 μL of complete medium containing 6 μg/mL BSH and 5 μg/mL polybrene. One milliliter of this mixture was added to each well, and cells were incubated for 8 h. The medium was then aspirated, the cells were washed with 1x dPBS, and fresh medium with 6 μg/mL BSH was added. Only transfected cells proliferated, effectively eliminating non‐transfected cells. Transfection was verified using a luminometer, fluorescence microscope, and In Vivo imaging device.

To assess transfection efficiency, 5 × 10^4^ transfected LoVo cells were seeded into six wells. After 24 h, the medium was aspirated, the wells were washed with 1x dPBS, and 1 μM d‐luciferin was added. Luminescence was measured using an In Vivo animal imaging device. Similarly, Balb/c athymic nude mice with xenografted LoVo cells were injected intraperitoneally with 10 μM d‐luciferin under anesthesia following tumor formation, and images were captured within 5 min using the IVIS (In Vivo Imaging System). Tumor regions (ROIs) were analyzed for luminescence (photons/sec) using IVIS Spectrum imaging software (Perkin‐Elmer, USA).

### 2.4 Cytotoxicity

TQ concentrations 2.5–100 μM, 5‐FU concentrations 0.039–25 μM, and various combined concentrations were added to 1 × 10^4^ cells per well, seeded on opaque white 96 plates, and the cells were incubated for 24 h. After 24 h, ATP solution was added, and luminescence measurements were conducted using a multi‐plate reader within 5 min (Varioskan Flash Multimode Reader, Thermo Scientific, USA).

#### 2.4.1 Combination Index and Synergism Analysis

The pharmacological interaction between TQ and 5‐FU, whether synergistic, additive, or antagonistic, was comprehensively assessed using the Chou‐Talalay method, a widely recognized approach for analyzing drug combination effects based on the median‐effect principle. The CI values were calculated using the Compusyn software, where CI < 1, CI = 1, and CI > 1 indicate synergism, additive effect, and antagonism, respectively. Dose‐effect curves, median‐effect plot, and CI‐Fraction Affected (Fa) curves were generated to evaluate the interaction between TQ and 5‐FU.

### 2.5 Intracellular Reactive Oxygen Species (ROS)

Cell lines were treated with TQ concentrations (2.5–100 μM), 5‐FU concentrations (0.039‐25 μM), and their combination for 24 h. Following treatment, the medium was washed with 1x dPBS. After washing, the plate was incubated at 37°C for 30 min with 5 µM H_2_DCF‐DA in each well. The intracellular ROS levels were measured using a fluorimeter (Multi‐Mode Reader, Thermo Scientific, Waltham).

### 2.6 Mitochondrial Membrane Potential (MMP)

The MMP was measured using a DiOC_6_(3) (3,3′‐dihexyloxacarbocyanine iodide) molecular fluorescence probe in flow cytometry. After 24 h incubation with TQ, 5‐FU, and the combination group, the medium was removed, and the cells were washed and incubated with 40 nM DiOC_6_(3) at 37°C for 15 min, followed by a final wash with 1xdPBS. The cells were then analyzed using flow cytometry (FACS Canto II, Becton Dickinson) (λexc\λem:484/501 nm) [14, 15].

### 2.7 Intracellular Glutathione

The glutathione (GSH) levels in LoVo‐ and CCD‐18Co cell lines were measured using a GSH/GSSG‐Glo kit (Promega, USA). The cell lines were seeded into opaque‐white 96‐well plates and treated with TQ concentrations (2.5–100 μM) for 24 h. The medium was then discarded, and 50 μL glutathione reagent was added to lyse the cells, followed by incubation at room temperature for 5 min on a shaker. Subsequently, 100 μL Luciferin reagent was added, and luminescence was measured using a Multimode Flash Reader (Thermo, Waltham, USA). Relative luminescence (RLU) was calculated as the control.

### 2.8 Genotoxicity

DNA damage was quantified using alkaline single‐cell electrophoresis, commonly referred to as the comet assay, as originally described by Singh et al. [16]. The effects of TQ on LoVo‐Luc and CCD‐18Co cells have been previously detailed [17]. For this assay, LoVo‐Luc and CCD‐18Co cells were seeded in 6‐well plates at a density of 75 × 10^3^ cells per well and treated with concentrations below IC_50_ of TQ. After 24 h of treatment, the cells were collected, centrifuged at 500 x *g* for 5 min, and the supernatant was discarded. The cell pellet was resuspended, and 10 µL of cell suspension was mixed with 85 µL of 0.65% low‐melting‐point agarose. This mixture was spread onto microscope slides pre‐coated with 1% normal melting point agarose and immediately placed on ice to solidify. The slides were incubated in lysis solution, which efficiently lyses cells and removes proteins, leaving behind nucleoids containing supercoiled loops of DNA. This process was carried out at +4°C for 4 h to ensure complete lysis. After lysis, the slides were incubated in electrophoresis buffer at +4°C for 40 min to allow for DNA unwinding and the expression of alkali‐labili sites and DNA strand breaks. Electrophoresis was performed at 26 V (300 mA) for 25 min at +4°C. The slides were washed in neutralization buffer and fixed with ethanol. For visualization, the DNA was stained with 2 μg/mL ethidium bromide, and images were captured under a fluorescence microscope (Nikon Eclipse Ts2). Both the migration and stretching of DNA from the nuclear core contributed to increased DNA damage [18]. The percentage of DNA in the comet tail (tail DNA %) was used as an indicator of DNA damage and was quantified using Comet Assay IV analysis software (Instem Group of Companies, UK).

### 2.9 Apoptosis

#### 2.9.1 Acridine Orange/Ethidium Bromide (AO/EB)

Cells seeded in six wells were harvested with trypsin‐EDTA 24 h after treatment at concentrations below IC_50_. The cells were washed with 1xdPBS and centrifuged at 500 x *g* at +4 ° C for 5 min. After centrifugation, the supernatant was discarded. Cell pellets (10 μL) were combined with 10 μL of acridine orange/ethidium bromide solution (100 μg/mL acridine orange + 100 μg/mL ethidium bromide) on an empty slide, and the coverslips were sealed. Subsequently, images were examined and captured using a fluorescence microscope (Leica DM 1000, Germany). Three replicates at each concentration, and approximately 100 randomly recorded cells were counted.

#### 2.9.2 Annexin V‐FITC Analysis

Apoptosis analysis using flow cytometry was performed according to the package insert of the Annexin V‐FITC Apoptosis Assay Kit (EBioscience BMS500F1, USA). Briefly, cells seeded in six wells were harvested using trypsin‐EDTA 24 h after treatment at concentrations below the IC_50_. Cells were washed with 1xdPBS and incubated with 195 μL 1x binding buffer and 5 μL annexin V‐FITC dye for 10 min at room temperature. After adding 200 μL of 1x binding buffer and centrifuging at 1500 x *g* at +4 ° C for 5 min, the supernatant was discarded. The pellet was resuspended in 190 μL of 1x binding buffer, and 10 μL of propidium iodide dye was added. The cells were vortexed and subsequently analyzed by flow cytometry (FACS Canto II, Becton Dickinson, USA) (λexc\λem:488/525 nm).

#### 2.9.3 Quantification of Apoptotic Protein Expressions by Western blot

Protein expression was analyzed by western blot analysis to determine the effects of TQ, 5‐FU, and the combination treatments on proapoptotic and antiapoptotic proteins. The protocol was adapted with slight modifications from the method described by Peiris et al. [19]. Primary antibodies (Bax, Bcl‐2, Caspase‐3, Caspase‐9, and β‐actin) and secondary antibodies (anti‐mouse IgG and anti‐rabbit IgG) were diluted 1:2000. After incubation with the antibodies, luminol solution (Pierce ECL Western blot analysis Substrate, Thermo, USA) was added before imaging the proteins on the membrane. Images were captured within 1 h using a Fusion Fx5 device (Vilbert Laumart, France).

### 2.10 Xenograft Colorectal Cancer Model; In Vivo

The ethics committee approval number 2017‐13 was obtained from the XXX Experimental Animal Ethics Committee. Male BALB/c athymic nude mice, 20‐25 grams, 8‐10 weeks old, were obtained from the Bezmialem Vakif University Experimental Animals Transgenic Laboratory. Nude mice were used in this study because they have a natural immunodeficiency that allows human cancer cells to be engrafted without additional immunosuppression. The animals were randomly divided into five groups, each containing six subjects (*n* = 6): Group I served as the negative control (healthy animals without cancer cell transplantation with xenograft), Group II as the positive control (intraperitoneal physiological saline) was given every other day after cancer cell injection with xenograft), Group III received TQ, Group IV was treated with 5‐FU, and Group V received a combination of TQ and 5‐FU. The mice were injected subcutaneously with 1.5 × 10^6^ transfected LoVo cells in dPBS. Mice were administered 1 mg/mL d‐luciferin 1 week after the injection, and controlled with IVIS. The ROI values were recorded.

#### 2.10.1 Treatment Procedure

Curative treatment was initiated 3 weeks after injection. TQ was administered at 10 mg/kg every other day, while 5‐FU was administered at 12.5 mg/kg every other day, with the same concentrations given in combination to the animals every other day. To examine tumor development, the animals were anesthetized using ketamine (30 mg/kg body weight) 4 days after injection, and 1 mg/mL d‐Luciferin was administered intraperitoneally. Images were captured within 10 min, and the ROI values were recorded. The tumor sizes were measured bidirectionally using a caliper before the animals were placed in the cages.

#### 2.10.2 Biochemical Analyzes

Before the xenograft procedure after sacrification, whole blood samples were collected from the rats by the intracardiac puncture in a lithium heparin tubes, and plasma was separated by centrifugation at 3000 × *g* for 10 min. Total antioxidant status (TAS), total oxidant status (TOS), thiol‐disulfide homeostasis inflammation markers, and growth factors (TGFβ1 and VEGF‐α) were measured in plasma samples.

##### 2.10.2.1 Oxidative Stress Analyzes

Methods developed by Erel were used to measure total thiol, native thiol homeostasis, TAS, and TOS [20, 21, 22]. Oxidative stress levels were measured using photometric method with commercially purchased kits (Total thiol, Rel assay HV21817; native thiol, Rel assay HN2101N).

The oxidative stress index (OSI) and disulfide level was calculated using the formulas:

OSI=TOS/TAS

Disulfide=(TT−NT)/2

##### 2.10.2.2 Tissue Analysis

Tumor tissues were homogenized with ceramic balls for biochemical analysis, homogenized for 1 min in 1xPBS buffer, and then centrifuged at 10,000 × *g* for 30 min at +4 °C [23].

#### 2.10.3 Tissue Protein Expression Analyzes

Protein quantification was performed using the Bradford method, which involves aspiration of the supernatant [24]. Expression of pro‐ and antiapoptotic proteins in cells was determined by Western blot analysis following a previously described procedure [14].

#### 2.10.4 Transforming Growth Factor Beta 1 (TGFβ1) and Vascular Endothelial Growth Factor A (VEGF‐α) ELISA

After protein determination in tissue homogenates, TGFβ1 (E‐EL‐M0051, Elabscience, USA) and VEGF‐α (E‐EL‐M1292, Elabscience, USA) levels were measured by photometric analysis using commercially purchased mouse ELISA kits.

### 2.11 Statistical Analysis

All In Vivo and In Vitro data are presented as the mean ± standard deviation (mean ± SD) of three replicates. Descriptive statistics, including means and SD, were calculated to summarize the data. To determine the statistically significant differences among the groups, a one‐way ANOVA was conducted. Pearson's correlation analysis was used to assess the relationships between variables. Post‐hoc analyses (e.g., Tukey's test) were used to compare the parameters among the various groups. Statistical significance was defined as *p* < 0.05. Additionally, nonlinear regression analysis was used to calculate the IC_50_ values for TQ, 5‐FU, and their combination in the cell lines. All statistical analyzes were carried out using SPSS for Windows software package (version 22, Chicago, IL, USA).

## 3 Results

### 3.1 Transfection, Cell Viability, and Intracellular ROS

**Figure 1:**
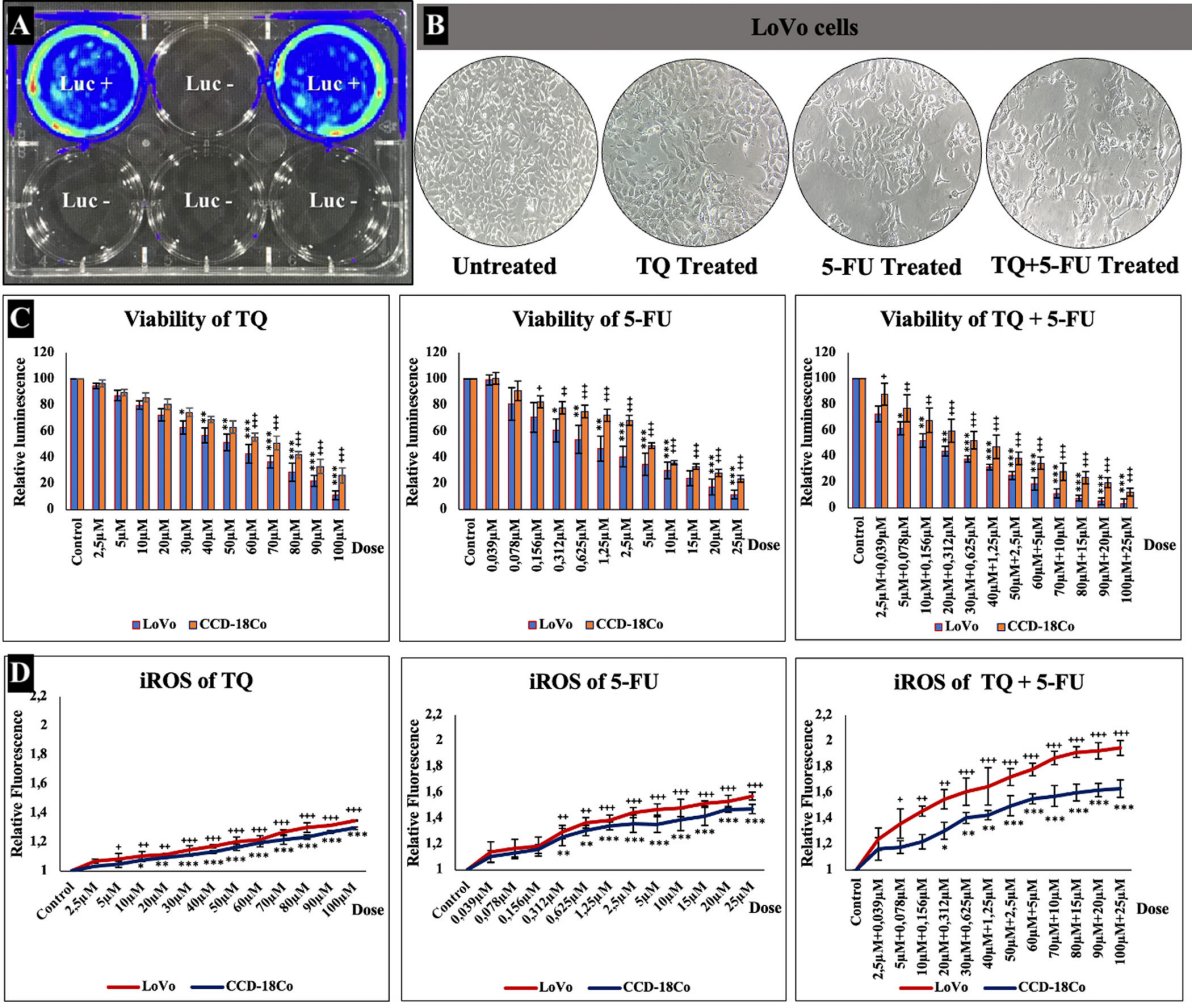
(A) Cell transfection control by In Vivo Imaging System (IVIS); To control the transfected cells, 1 μM d‐luciferin was prepared in dPBS and added onto the cells. Luminometric radiation was detected in LoVo‐Luc cells at the first and third wells in contrast to non‐transfected cells on the In Vivo Imaging System (IVIS)device within 5 min. (B) The morphology of TQ‐,5‐FU‐, and TQ+5‐FU‐ treated cells. (C) Viability of LoVo human colorectal cancer cells and healthy cells CCD‐18Co. (D) Effect of thymoquinone (TQ), 5‐Fluorouracil (5‐FU), and combined treatment with TQ and 5‐FU on intracellular ROS, following incubation of cells with 2.5–100 µM for 24 h. Statistically significant differences in the healthy cell line CCD‐18Co, *p < 0.05, **p < 0.01, ***p < 0.001; differences in the human colorectal cancer cell line LoVo ^+^ *p* < 0.05, ^++^ *p* < 0.01, and ^+++^ *p* < 0.001 compared to the control group. Data are presented as the mean ± standard deviation (mean ± SD) of three replicates.

Cells were seeded into six wells after transfection under the control of luciferase transfection. After 24 h of incubation, cells were visualized using an IVIS instrument. Wells 1 and 3 were found to be Luc‐positive, whereas the others were Luc‐negative (Figure 1A).

To examine the cytotoxic effects on CCD‐18Co and LoVo cell lines after 24 h of incubation, a luminometric method was employed to measure intracellular ATP levels. The results indicated a significant increase in cytotoxicity and a concomitant decrease in cell viability (*p* < 0.001) with increasing concentrations of all substances tested (Figure 1C). The IC_50_ value was calculated as 75.003 µM for TQ, 0.956 µM for 5‐FU, and 12.543 µM for the combination therapy.

When TQ was administered to the cells, ROS levels increased significantly compared to the control groups (*p* < 0.001). Although cancer cells typically exhibit higher metabolic activity and ROS production than healthy cells, TQ, 5‐FU, and their combination treatment led to further increases in ROS levels (Figure 1D).

### 3.2 Dose–Response, Combination Index, and Median Effect Analyses

**Figure 2:**
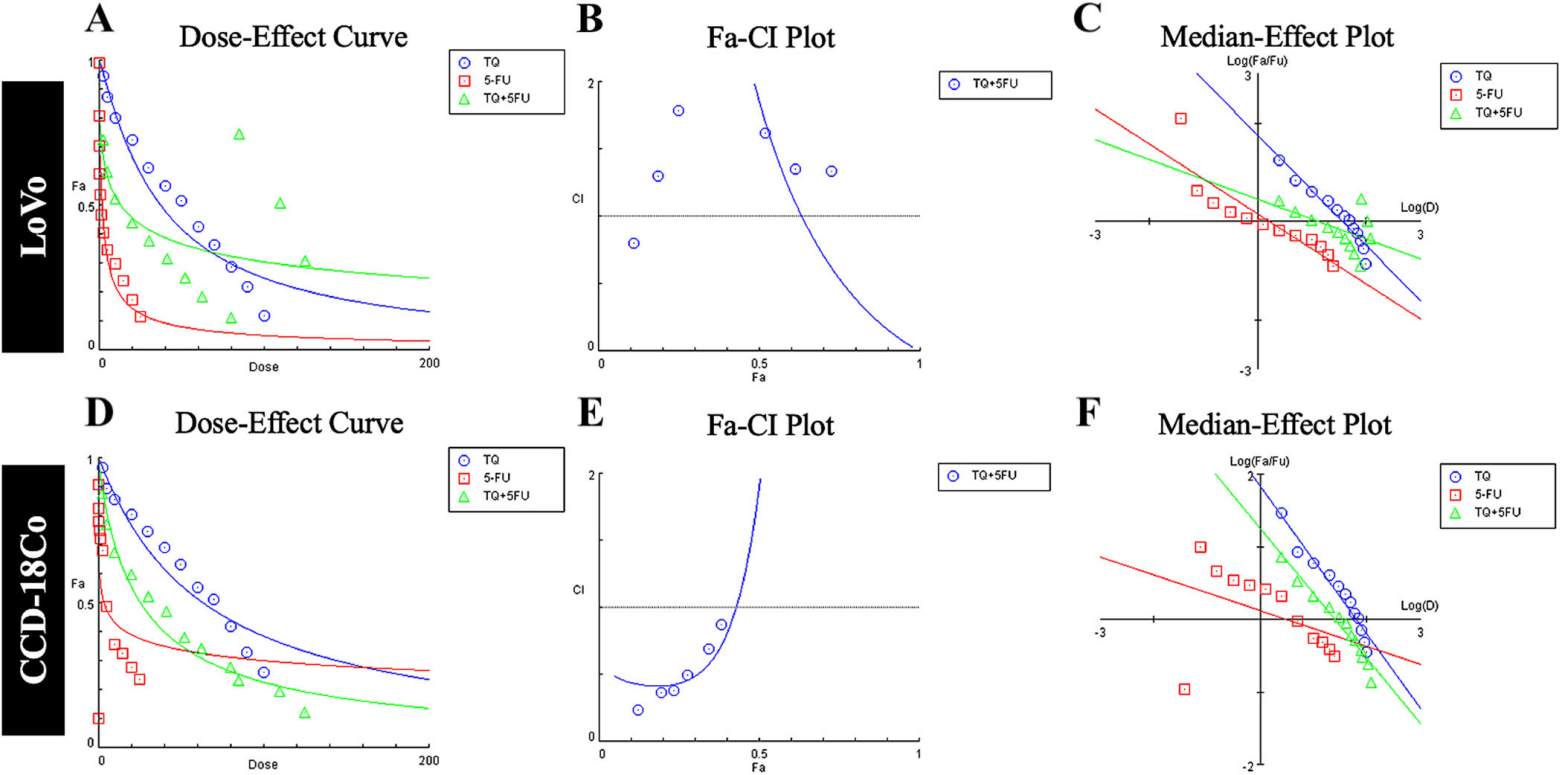
Simulated plots for analyzing pharmacodynamic interactions were generated using CompuSyn software. (A, D) dose–response curves of thymoquinone (TQ) and 5‐fluorouracil (5‐FU) alone and in combination. The x‐axis represents the dose, while the y‐axis shows the fraction of affected cells (Fa). (B, E) The Fa‐CI Plots depict the Combination Index (CI) as a function of the fraction affected (Fa). A CI < 1 indicates synergy, CI = 1 suggests additive effects, and CI > 1 denotes antagonism. (C, F) The Median‐Effect Plots illustrate the dose–response relationships for TQ, 5‐FU, and their combination in LoVo and CCD18Co cells. The x‐axis represents the logarithmic dose, while the y‐axis corresponds to the logarithm of the ratio between affected and unaffected fractions (log10(Fa/(1 − F*a*))).

The cytotoxic effects of TQ, 5‐FU, and their combination on LoVo and CCD‐18Co cell lines were analyzed using median‐effect plots and CI‐Fa curves based on the Chou‐Talalay method (Figure 2). Synergistic interactions (CI < 1) were observed at low to moderate exposure levels (Fa > 0.75). At higher exposure levels (Fa = 0.5), CI values exceeded 1, suggesting additive or antagonistic effects. The median effect dose and slope were calculated to evaluate dose–response dynamics and interactions between the two agents. In LoVo cells, dose‐effect plots showed that in addition to the independent effects of TQ and 5‐FU, combination (TQ + 5‐FU) treatment increased cell response. Combination therapy shows a synergistic effect while increasing the cell response rate at low doses. This suggests that combination therapy may improve efficacy and offer an effective treatment strategy at lower toxic doses.

Combination therapy has shown potential for enhanced anticancer efficacy at lower doses, reducing the required 5‐FU concentrations and minimizing off‐target toxicity, demonstrating its potential therapeutic advantage in colorectal cancer treatment.

### 3.3 GSH Levels

The effects of TQ and 5‐FU on glutathione levels in LoVo and CCD‐18Co cells are presented in Table 1. Both TQ and 5‐FU, when administered alone or in combination, caused a dose‐dependent decrease in glutathione levels in both cell lines. In LoVo cells, a significant decrease in glutathione levels was observed when either agent was administered alone, and this decrease became more pronounced with combination treatment. Particularly, the combination of 30 µM TQ + 0.625 µM 5‐FU and higher concentrations resulted in a significant reduction in glutathione levels in LoVo cells at (*p* < 0.001). Although reductions were less severe compared to those in cancer cells, suggesting that the combination treatment has a more selective effect against cancer cells.

**Table 1:** Effect of TQ and 5‐FU on glutathione levels of human colorectal cancer cells LoVo, and healthy cells CCD‐18Co.

TQ			5‐FU			Combined		
	CCD‐18Co	LoVo		CCD‐18Co	LoVo		CCD‐18Co	LoVo
Control	4.99	5.00	***Control***	4.98	5.00	***Control***	4.93	5.00
2.55 µM	4.92	4.62	***0,039 µM***	4.82	4.28	***2.5 µM*** + ***0,039 µM***	4.82	4.14
5 µM	4.86	4.41	***0,078 µM***	4.65	4.15	***5 µM*** + ***0,078 µM***	4.62	3.87
10 µM	4.63	4.30	***0,156 µM***	4.33	3.99	***10 µM*** + ***0,156 µM***	4.32	3.67
20 µM	4.47	4.05	***0.312 µM***	4.21	3.85	***20 µM*** + ***0,312 µM***	4.06	3.13
30 µM	4.19	3.97	***0.625 µM***	3.99	3.46	***30 µM*** + ***0,625 µM***	3.69*	2.94^**+**^
40 µM	3.92*	3.63^**+**^	***1.25 µM***	3.65*	3.19^**+**^	***40 µM*** + ***1,25 µM***	3.54**	2.62^**++**^
50 µM	3.70**	3.33^**++**^	***2.5 µM***	3.49**	3.07^**++**^	***50 µM*** + ***2.5 µM***	3.41**	2.32^**++**^
60 µM	3.61**	3.25^**++**^	***5 µM***	3.11**	2.65^**++**^	***60 µM*** + ***5 µM***	3.07***	2.04^**+++**^
70 µM	3.35**	3.00^**++**^	***10 µM***	2.84***	2.35^**+++**^	***70 µM*** + ***10 µM***	2.72***	1.87^**+++**^
80 µM	3.13***	2.82^**+++**^	***15 µM***	2.67***	2.02^**+++**^	***80 µM*** + ***15 µM***	2.50***	1.61^**+++**^
90 µM	2.82***	2.37^**+++**^	***20 µM***	2.56***	1.96^**+++**^	***90 µM*** + ***20 µM***	2.41***	1.49^**+++**^
100 µM	2.70***	2.24^**+++**^	***25 µM***	2.33***	1.67^**+++**^	***100 µM*** + ***25 µM***	2.32***	1.32^**+++**^

*Note:* For differences in LoVo cells ^+^ *p* < 0.05, ^++^ *p* < 0.01, ^+++^ *p* < 0.001; and in CCD‐18Co cells *p < 0.05, **p < 0.01, ***p < 0.001 values are regarded as statistically significant.

Abbreviations: 5‐FU, 5‐Fluorouracil; SD, Standard deviation; TQ, Thymoquinone.

### 3.4 MMP

The effects of TQ and 5‐FU on MMP are analyzed in Table 2. Increasing combination concentrations of TQ and 5‐FU below IC_*50*_ values in both cell lines caused a statistically significant decrease in MMP. In LoVo cells, administration of TQ and 5‐FU alone and in combination led to a significant dose‐dependent reduction in MMP. Specifically, the combination of 20 µM TQ + 0.312 µM 5‐FU and higher doses led to a significant decrease in MMP in LoVo cells (*p* < 0.001).

**Table 2:** Effect of TQ and 5‐FU on mitochondrial membrane potential: Increasing Combined concentrations below IC_50_ values decrease MMP in all two cell lines statistically significantly.

TQ			5‐FU			Combined		
	CCD‐18Co	LoVo		CCD‐18Co	LoVo		CCD‐18Co	LoVo
Control	92.00	93.00	***Control***	93.00	95.00	***Control***	94.00	95.00
5 µM	88.00	8000	***0,078 µM***	81.00	76.00	***5 µM*** + ***0,078 µM***	77.00*	71.00^**+**^
10 µM	77.00*	72.00^**+**^	***0,156 µM***	71.00*	65.00^**+**^	***10 µM*** + ***0,156 µM***	62.00**	58.00^**++**^
20 µM	71.00**	67.00^**++**^	***0,312 µM***	63.00**	58.00^**++**^	***20 µM*** + ***0,312 µM***	53.00***	49.00^**+++**^
40 µM	66.00***	60.00^**+++**^	***1,25 µM***	60.00***	45.00^**+++**^	***40 µM*** + ***1,25 µM***	44.00***	38.00^**+++**^
60 µM	62.00***	55.00^**+++**^	***5 µM***	55.00***	33.00^**+++**^	***60 µM*** + ***5 µM***	32.00***	16.00^**+++**^

*Note:* For differences in LoVo cells ^+^ *p* < 0.05, ^++^ *p* < 0.01, ^+++^ *p* < 0.001; and in CCD‐18Co cells *p < 0.05, **p < 0.01, ***p < 0.001 values are regarded as statistically significant.

Abbreviations: 5‐FU, 5‐Fluorouracil; SD, Standard deviation; TQ, Thymoquinone.

### 3.5 Apoptosis, Cell Protein Expression, and DNA Damage

After 24 h of treatment with sub‐IC_50_ concentrations, early and late apoptosis, as well as necrosis, increased dose‐dependently, with combination therapy further enhancing the levels of apoptotic and necrotic cells. The AO/EB double staining method revealed a dose‐dependently increase in apoptosis (*p* < 0.001).

**Figure 3:**
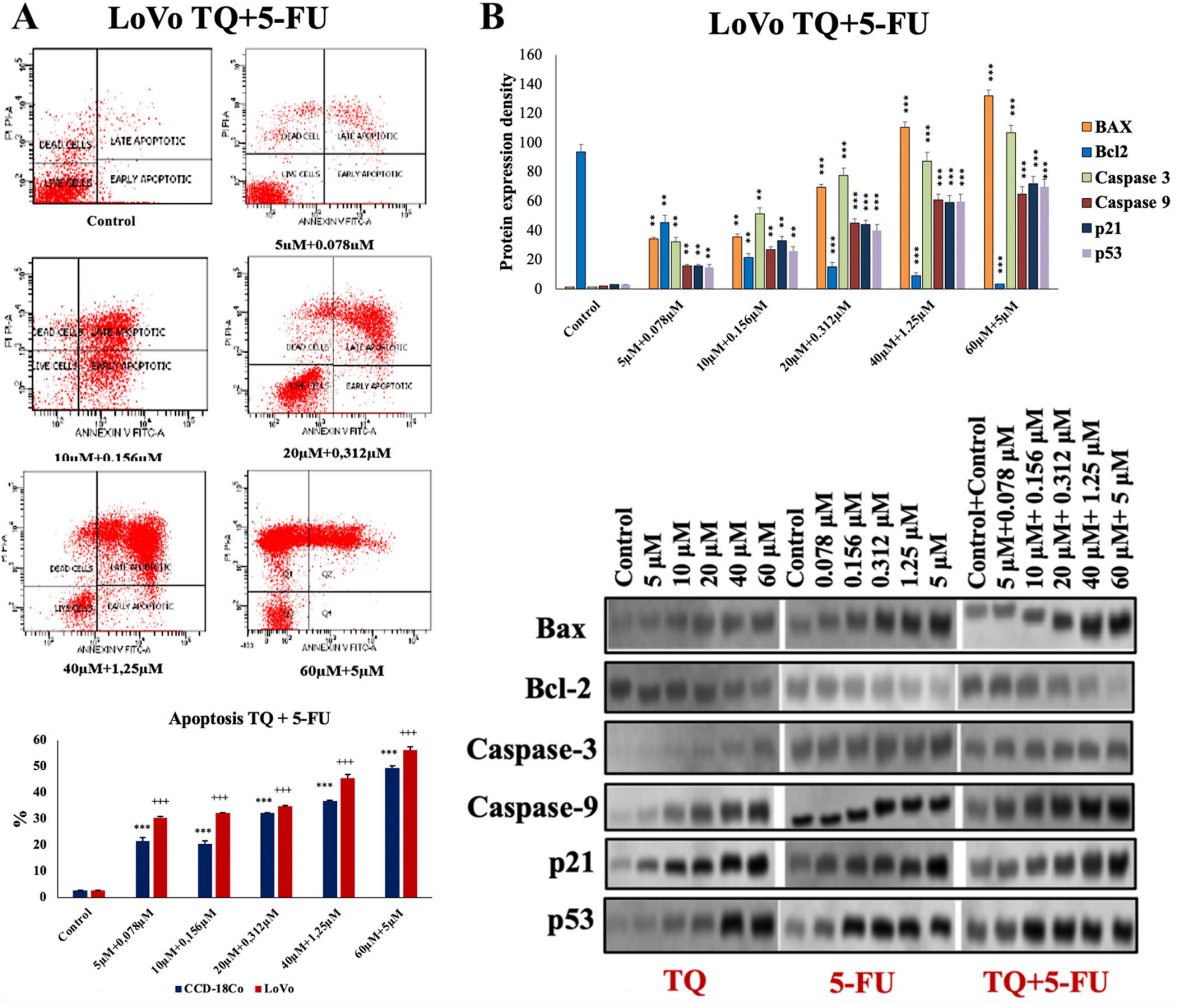
Effect of thymoquinone (TQ) and 5‐Fluorouracil (5‐FU) on (A) apoptosis by Annexin V‐FITC Analysis and Acridine Orange/Ethidium Bromide stain. For differences in LoVo cells, *p < 0.05, ** for *p* < 0.01, *** for *p* < 0.001; and in CCD‐18Co cells, ^+^ *p* < 0.05, ^++^ *p* < 0.01, ^+++^ *p* < 0.001 were regarded as statistically significant. (B) Cell protein expression of Bcl‐2, Caspase 3, Caspase 9, p21, and. P53 proteins in LoVo cells. Data are presented as the mean ± standard deviation (mean ± SD) of three replicates.

Protein expression analysis via western blot analysis in CCD‐18Co and LoVo cells demonstrated an increase in Bax, p53, p21, Caspase‐3, and Caspase‐9 expression with higher concentrations, while Bcl‐2 decreased (*p* < 0.001), see Figure 3.

**Figure 4:**
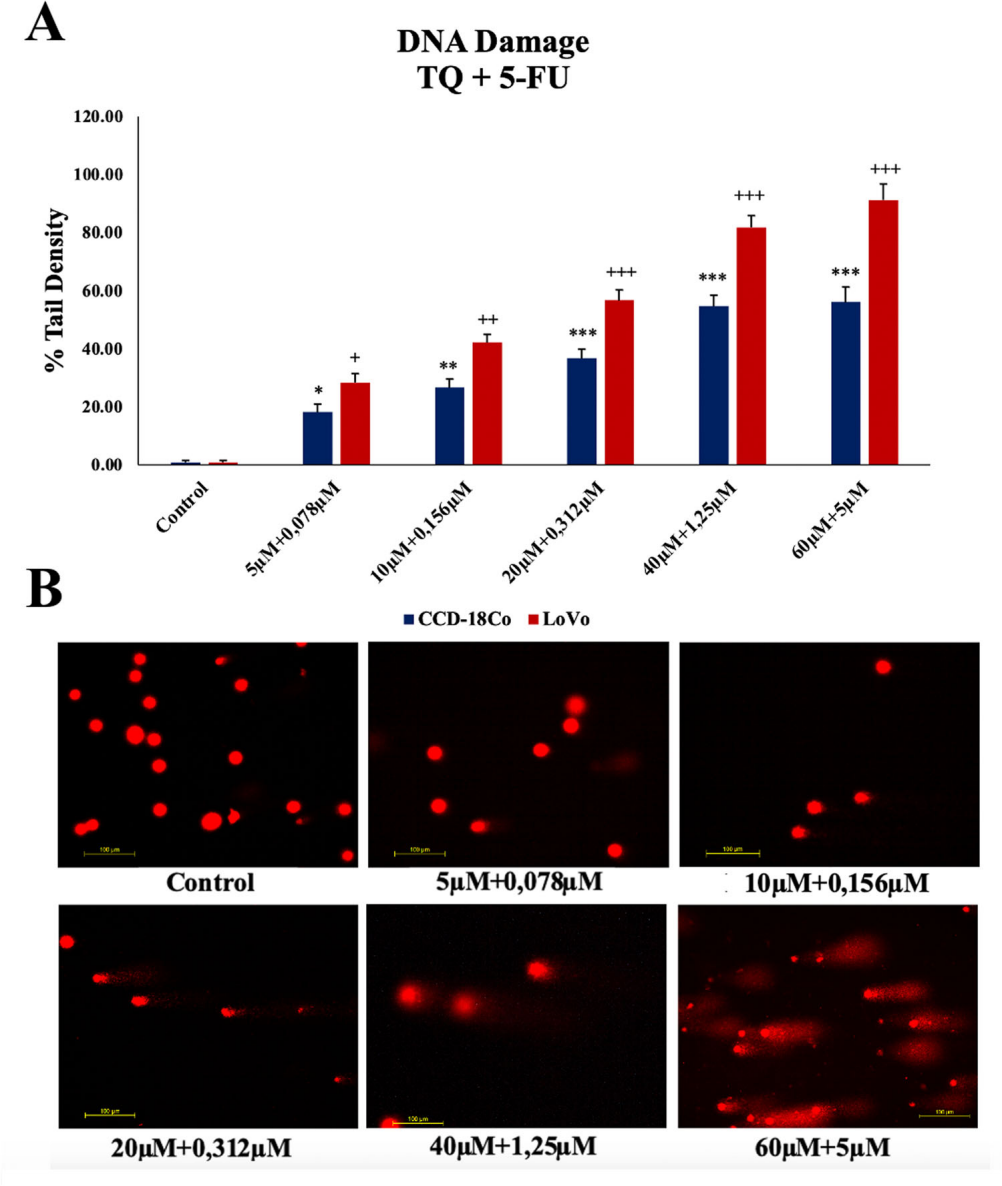
Effect of combined therapy on DNA damage: In the experiment with comet assay, (A) DNA damage (comet tail moment) was statistically significantly increased in both healthy and cancer cells at concentrations below IC_50_. (B) The representative immunofluorescence image of DNA damage of combination concentration thymoquinone (TQ) and 5‐Fluorouracil (5‐FU). For differences in LoVo cells, *p < 0.05, ** for *p* < 0.01, *** for *p* < 0.001; and in CCD‐18Co cells, ^+^ *p* < 0.05, ^++^ *p* < 0.01, ^+++^ *p* < 0.001 were regarded as statistically significant.

DNA damage, as indicated by varying concentrations of TQ, 5‐FU, and combination treatment at IC_50_ concentrations, correlated with increased DNA tail density. Cancer cells exhibited higher DNA tail density due to genetic instability, elevated ROS levels, and reduced GSH. TQ, 5‐FU, and combination treatments further increased DNA tail densities (Figure 4). DNA damage in cancer cells was four times higher with TQ and 5‐FU combination than TQ alone and 1.5 times higher than with 5‐FU alone.

### 3.6 TQ and 5‐FU on the Levels of Oxidative Stress in the Serum

Oxidative stress biomarkers measured in the serum of nude mice following TQ and 5‐FU treatment were evaluated, see Table 3. Compared to the positive control group, TOS levels were significantly elevated in the TQ and 5‐FU treatment groups. Particularly, significant increases in TOS levels were observed in the 5‐FU and TQ + 5‐FU combination treatment groups (*p* < 0.01) compared to the positive control group. Regarding TAS levels, while a significant decrease was observed in the positive control group, TQ treatment partially prevented this decrease. However, TAS levels remained low in 5‐FU and combination groups, similar to the positive control group. When evaluating OSI, a significant increase was observed in the positive control group. OSI values were also significantly higher in the TQ and 5‐FU treated groups, and the highest OSI values recorded in the combination treatment group.

**Table 3:** Oxidative stress biomarkers in parameters in the serum of nude mice after the treatment with TQ and 5‐FU.

Oxidative stress biomarkers					
	Negative Control	Positive Control	TQ	5‐FU	TQ + 5‐FU
	Mean ± SD	Mean ± SD	Mean ± SD	Mean ± SD	Mean ± SD
Total Oxidant Status µmol H_2_O_2_ Eq./L	6.25 ± 1.01	12.61 ± 1.38***	8.93 ± 1.03^**+**^	15.87 ± 0.97^**++**^	9.76 ± 2.14^**++**^
Total Antioxidant Status mmol Trolox Eq./L	1.56 ± 0.13	0.76 ± 0.10***	1.06 ± 0.08^**+**^	0.62 ± 0.06^**+**^	0.90 ± 0.06
Oxidative Stress Index AU	4.07 ± 1.03	16.87 ± 3.62***	8.47 ± 1.10^**++**^	25.57 ± 2.71^**++**^	10.65 ± 1.75^**++**^
Total Thiol µM	645.56 ± 30.22	449.79 ± 36.82***	515.35 ± 11.06^**+**^	393.30 ± 11.21^**+**^	478.60 ± 21.33^**+**^
Native Thiol µM	570.77 ± 16.78	321.05 ± 23.04***	413.37 ± 14.71^**+**^	155.72 ± 25.77^**+**^	356.30 ± 12.21^**+**^
Disulfide µM	37.42 ± 18.43	64.37 ± 15.14*	50.99 ± 7.33^**+**^	118.78 ± 16.40^**+**^	61.15 ± 13.73^**+**^

*Note:* Negative control (healthy animals without cancer cell transplantation with xenograft), and positive control (intraperitoneal physiological saline). Statistically significant differences of relative values in treatment groups, *p < 0.05, **p < 0.01, ***p < 0.001; ^+^ *p* < 0.05, ^++^ *p* < 0.01, ^+++^ *p* < 0.001.

Abbreviations: 5‐FU, 5‐Fluorouracil; SD, Standard deviation; TQ, Thymoquinone.

Analysis of total thiol, native thiol, and disulfide levels revealed significant decreases in thiol levels and increases in disulfide levels in the positive control group due to oxidative stress (*p* < 0.05). While partial improvements in thiol levels were observed in the TQ and 5‐FU treatment groups, the increase in disulfide levels persisted.

#### 3.6.1 Pro‐inflammatory Cytokine Markers

The levels of inflammatory markers (IL1β, IL6, TNF‐α, and HsCRP) and growth factors (VEGF‐α and TGFβ1) were significantly higher compared to the negative control group. In comparison to the positive control group, 5‐FU treatment increased the levels of all inflammatory cytokines. However, due to the systemic antioxidant effect of TQ, inflammation was significantly reduced in all treatment groups compared to the positive control group (Table 4).

**Table 4:** Inflammatory and angiogenesis biomarkers in parameters in the serum of nude mice after the treatment with TQ and 5‐FU.

Biomarkers						
		Negative Control	Positive Control	TQ	5‐FU	TQ + 5‐FU
		Mean ± SD	Mean ± SD	Mean ± SD	Mean ± SD	Mean ± SD
Inflammatory	**hs‐CRP** pg/mL	851.14 ± 94.68	1479.01 ± 207.93***	1087.83 ± 76.50^**+**^	1820.13 ± 120.62^**+**^	1275.50 ± 190.08
	**IL‐1β** pg/mL	140.36 ± 27.83	293.53 ± 25.18***	201.25 ± 28.44^**+**^	344.50 ± 27.74^**+**^	243.32 ± 20.41^**+**^
	**IL‐6** ng/L	58.18 ± 10.61	114.64 ± 22.07***	80.70 ± 17.57^**+**^	154.19 ± 15.88^**++**^	94.94 ± 13.37^**+**^
	**TNF‐α** ng/L	272.15 ± 29.79	495.95 ± 40.20***	368.45 ± 46.54^**+**^	580.24 ± 43.28^**++**^	427.86 ± 20.11^**+**^
	**TGFβ1** ng/L	219.28 ± 26.70	732.35 ± 78.57***	583.10 ± 70.37^**+**^	502.43 ± 47.37^**++**^	405.69 ± 32.14^**+++**^
Angiogenesis	**VEGF‐α** ng/L	320.98 ± 24.68	852.53 ± 117.59***	731.80 ± 100.10^**+**^	629.42 ± 142.22^**++**^	492.56 ± 62.06^**+**^

*Note:* Negative control (healthy animals without cancer cell transplantation with xenograft), and positive control (intraperitoneal physiological saline). Statistically significant differences of relative values in inflammatory biomarkers, *p < 0.05, **p < 0.01, ***p < 0.001; ^+^ *p* < 0.05, ^++^ *p* < 0.01, ^+++^ *p* < 0.001.

Abbreviations: hs‐CRP, high‐sensitivity C‐reactive protein; IL‐1β, Interleukin‐1 beta; IL‐6, Interleukin‐6; SD, Standard deviation; TQ, Thymoquinone; TNF‐α, Tumor necrosis factor‐alpha; TGFβ1, Transforming Growth Factor Beta 1; VEGF‐α, Vascular Endothelial Growth Factor A; 5‐FU, 5‐Fluorouracil.

The levels of growth factors such as VEGF‐α and TGFβ1 were significantly higher in the positive control group compared to the negative control group. TQ treatment significantly decreased these levels in the 5‐FU and combination treatment groups relative to the positive control group.

#### 3.6.2 Effects of Combined Therapy of TQ and 5‐FU on Tissue Levels of Pro‐inflammatory Cytokine Markers and Protein Expression Levels

**Figure 5:**
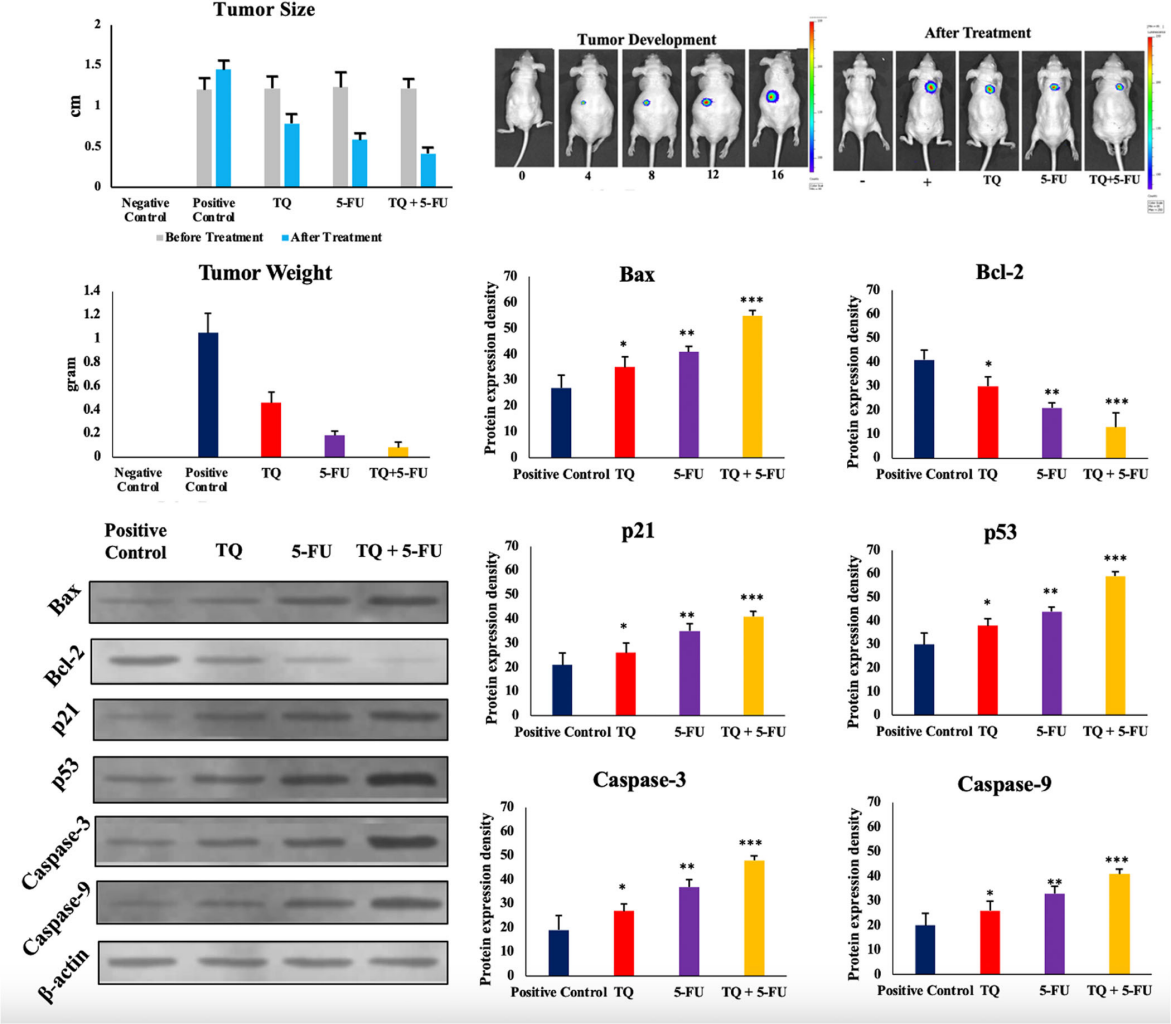
In vivo xenograft colorectal cancer model: In Vivo Imaging System (IVIS) images of tumor sizes of the tumor development process on different days according to groups and **t**umor tissue Bax, Bcl‐2, Caspase‐3, Caspase‐9, p21, and p53 protein expression: All proteins except Bcl‐2 increased significantly in the TQ, 5‐FU, and combined treatment groups compared to the positive control group. The results were normalized to the housekeeping protein, β‐actin. *p < 0.05, ** for *p* < 0.01, *** for *p* < 0.001. Data are presented as the mean ± standard deviation (mean ± SD) of three replicates.

Tumor sizes were reduced in all treatment groups, with combined treatment showing higher efficacy compared to single treatments, both in terms of macroscopic observations and tumor weight. The expression of all proteins (Bax, p53, p21, Caspase‐3, and Caspase‐9), except the antiapoptotic protein Bcl‐2, was significantly increased in the TQ, 5‐FU, and combined treatments, respectively, compared to the control group. Bcl‐2 expression significantly decreased in the TQ, 5‐FU, and combined treatment groups (Figure 5). VEGF‐α and TGFβ1 levels were significantly higher in the positive control group than in the negative control group. TQ treatment significantly decreased these levels in the 5‐FU and combination treatment groups relative to the positive control group (Table 5).

**Table 5:** Pro‐inflammatory and angiogenesis biomarkers in parameters on tissue of nude mice after the treatment with TQ and 5‐FU.

Cytokine Markers					
		Control	TQ	5‐FU	TQ + 5‐FU
		Mean ± SD	Mean ± SD	Mean ± SD	Mean ± SD
Pro‐inflammatory	**IL‐1β**	494.26 ± 39.43***	434.38 ± 30.94	388.48 ± 19.72	332.36 ± 28.73
	**IL‐6** ng/L	571.08 ± 38.52***	500.78 ± 55.52^**+**^	454.59 ± 62.31^**++**^	385.48 ± 29.88^**+**^
	**TNF‐α** ng/L	1247.34 ± 71.37***	1116.05 ± 63.75^**+**^	1010.57 ± 78.44^**++**^	931.28 ± 59.97^**+**^
	**TGFβ1** ng/L	1710.81 ± 57.08***	1542.26 ± 96.32^**+**^	1384.68 ± 27.54^**++**^	1024.92 ± 68.85^**+++**^
Angiogenesis	**VEGF‐α** ng/L	1588.02 ± 144.71***	1421.11 ± 157.82^**+**^	1333.74 ± 81.24^**++**^	1153.43 ± 99.83^**+**^

*Note:* Statistically significant differences of relative values in inflammatory biomarkers, *p < 0.05, **p < 0.01, ***p < 0.001; ^+^ *p* < 0.05, ^++^ *p* < 0.01, ^+++^ *p* < 0.001.

Abbreviations: IL‐1β, Interleukin‐1 beta; IL‐6, Interleukin‐6; hs‐CRP, high‐sensitivity C‐reactive protein; SD, Standard deviation; VEGF‐α, Vascular Endothelial Growth Factor A; TGFβ1, Transforming Growth Factor Beta 1; TNF‐α, Tumor necrosis factor‐alpha; TQ, Thymoquinone; 5‐FU, 5‐Fluorouracil.

## 4 Discussion

The development of potential natural products and substances as alternative combined therapeutic strategies is currently fundamental for improving the prognosis and cure rate of CC. Various approaches involving combinations of plant‐derived drugs have been suggested to overcome therapeutic resistance to 5‐FU and minimize its side effects. TQ, a natural bioactive compound and the active ingredient of NS, is well‐known for its robust and pluripotent antitumor activity and continues to be an attractive candidate in cancer therapy [25, 26, 27]. In this study, we demonstrated that the In Vivo activity of 5‐FU could be increased by TQ in a colorectal cancer cell line and colorectal cancer model created using the xenograft method. The combined therapy showed more potent anticancer effect than monotherapy. Additionally, we observed increased apoptosis and genotoxicity in the combined treatment group. In Vivo studies showed that it reduced tumor size, inflammation, and vascularization and showed promising results. This study is particularly significant as it is the first of its kind in the literature, demonstrating the single and combined efficacy of TQ and 5‐FU in transfected colorectal cancer cells and in a xenograft model established after injecting these cells into nude mice.

Several studies have explored the efficacy of combining TQ with various chemotherapeutic agents to enhance treatment outcomes [28, 29]. Lei et al. showed that the combination of high‐dose TQ (50 μM) and 5‐FU (75 μg/ml) induced apoptosis [30]. In their 2019 study, Ndreshkjana et al. found that this combination was more effective than single treatment in HCT116 and HT29 cells and that the dose could be reduced [30]. TQ was found to negatively affect cell proliferation in colon cancer and lymphoma cells. In a study conducted by Acar et al. in 2015 in head and neck cancer cells, it was shown that the combination of TQ and 5‐FU was more cytotoxic than single treatment and doses could be reduced [31]. Our study confirms that the combination of TQ and 5‐FU is more effective at lower doses than monotherapy. This single and combined treatment significantly reduced cell viability in both LoVo and CCD18Co cells. Furthermore, the combination of TQ and 5‐FU showed synergistic cytotoxic effects on LoVo cells, while the effects on CCD‐18Co cells were more limited and often shifted towards additive or antagonistic interactions at higher exposure levels. These results highlight the therapeutic potential of TQ and 5‐FU in selectively targeting cancer cells while sparing normal fibroblast‐like cells.

Normal cellular oxygen metabolism constantly produces iROS, which are known to cause oxidative damage to biomolecules, leading to oxidative stress. This pathophysiological process can result in apoptosis, autophagy, hypertension, atherosclerosis, diabetes, neurodegenerative diseases, and cancer [32]. Previous cell culture studies have shown that 5‐FU increases the intracellular ROS levels in cancer cells [33]. Recent studies have used TQ to induce ROS production because of its pro‐oxidant properties in resistant tumors [34, 35]. Although there are many studies in which TQ and 5‐FU induced intracellular ROS and studies in which both were used in the same cell, iROS levels were not measured. The amount of intracellular ROS increased in single and combined treatments with increased TQ and 5‐FU. In the combination therapy, a higher ROS level was achieved at a low concentration, and a positive correlation was found between increased iROS levels and cytotoxicity. Our study is the first to show that ROS is the source of cytotoxicity, genotoxicity, and apoptosis after single and combined treatments. In addition, intracellular glutathione levels play a central role in cellular antioxidant defenses against free radicals. In an In Vivo study of Ince et al. found that cypermethrin‐induced mice had normal glutathione levels after treatment with TQ, which may have resulted from TQ‐mediated reduction of peroxidative activity between cells [36]. In our study, the amount of glutathione decreased with increasing concentrations of TQ and 5‐FU. Compared to single therapy, high glutathione inhibition appears at low concentrations in the combination therapy.

One of the most effective methods for killing cancer cells is severe damage to cellular DNA. Therefore, most antineoplastic drugs used to treat cancer act directly on DNA. Apoptotic pathways are activated, and the cell undergoes apoptosis when the DNA damage is too great to be repaired. 5‐FU acts on cellular RNA by inhibiting thymidylate synthase (TS) by adding UTP or FdUTP to DNA instead of the thymidinate triphosphates required for DNA synthesis [37]. In addition, it binds to DNA with high affinity and seriously affects its structure [38].

TQ, on the other hand, exerts cytotoxic, genotoxic, and apoptotic effects either by directly targeting DNA or by increasing intracellular ROS levels [39]. While TQ has been studied in combination with various antineoplastic agents, only two studies have focused on its combination with 5‐FU. In these studies, WNT affected β‐catenin, NF‐kB, COX‐2, iNOS, and VEGF expression. It induces DNA damage by increasing chemosensitivity [40]. In our study, DNA damage was increased in the single and combined treatment groups. In the combination therapy, the tail density of damaged DNA increased, while the concentration decreased. Increased intracellular ROS levels were the primary effect of DNA damage. Although increasing ROS levels increases cytotoxicity, it can also induce DNA damage and apoptosis.

Tumor growth relies on mechanisms activated by various signals generated by tumor cells [41]. TGF‐β1 and VEGF‐α inhibit apoptosis [42]. Our study examined VEGF‐α and TGF‐β1 levels in the blood of animals and resected tumor homogenates after treatment with TQ, 5‐FU, or their combination. We showed that these values increased significantly in the positive control group, which developed tumors, compared with the healthy negative control group. It was demonstrated that VEGF‐α and TGF‐β1 levels decreased significantly with treatment, and that the combined treatment was more effective.

Antineoplastic drugs, excluding surgery and immunotherapy, primarily induce apoptosis in cancer cells. Developers aim to activate or correct apoptotic mechanisms. All current cytotoxic anticancer therapies induce apoptosis. Recently, TQ has been shown to induce apoptosis via oxidative damage, though early studies highlighted its antioxidant chemopreventive properties [43, 44]. Numerous studies have demonstrated that 5‐FU induces apoptosis in various tumor cells, similar to other chemotherapeutic agents [45, 46]. In line with the literature, our study found that apoptosis rates increased with the combined treatment, as measured by fluorescence microscopy using AO/EB staining and flow cytometry using annexin V‐FITC staining. The combined treatment induced apoptosis in more cells than the single treatments. Additionally, in our study, the expression of the antiapoptotic protein Bcl‐2 decreased In Vitro, in line with the literature, with increasing TQ, 5‐FU, and combined treatment concentrations. The expression of p21, p53, caspase‐3, caspase‐9, Bax, and apoptotic proteins increased in all groups. In Vivo, when resected tumors are homogenized, and we look at the same apoptotic biomarkers, it has been observed that single and combined treatments suppress the antiapoptotic mechanism while activating the proapoptotic mechanism.

In this study, we evaluated the effect of the combination of TQ and 5‐FU on colorectal using In Vitro cell culture and In Vivo experimental studies using the IVIS system for the first time. No oral administration was used in this study because certain drugs can have altered bioavailability and pharmacokinetic properties with oral administration. Oral drugs must pass through the gastrointestinal tract, undergo intestinal absorption, and hepatic first‐pass metabolism, whereas an intravenous drug is assumed to enter the systemic circulation immediately, as they are not subject to absorption or first‐pass metabolism [47]. This approach provides more accurate results by allowing the drug to act directly on the tumor cells.

The IVIS system allows real‐time visualization of tumor structures and sizes within a living animal using bioluminescent or fluorescent markers. This method is increasingly used in medical and biological research. In this study, Luc‐labeled cells were used for In Vivo imaging. The advantage of In Vivo imaging of Luc‐labeled cells lies in the integration of the luciferase gene into the chromosomes. The luciferase gene is stably expressed even when cells divide and differentiate. Luciferin, which acts as a substrate for the luciferase enzyme, triggers luminescence. The intensity of the emitted signal was directly correlated with the number of cells transfected with luciferase. Our IVIS results demonstrated a significant reduction in tumor size 30 days after treatment with TQ, 5‐FU, or their combination. In addition, TQ caused an effective concentration of 5‐FU and decreased the 5‐FU‐induced side effects.

## 5 Conclusion

In our study, we have shown that TQ is a potent anticancer molecule that regulates numerous molecular mechanisms, thereby demonstrating its potential as an excellent therapeutic agent for the prevention and treatment of cancer. While TQ currently lacks an oral or intravenous form, its combination with 5‐FU, a drug commonly used in conventional treatments, could be applied following the development of new viable formulations. Nevertheless, further research is required to investigate the effect of TQ on cancer in greater detail. The findings of this study show that, although a single treatment with TQ or 5‐FU has a pronounced anticancer effect in this model, their combination exhibits a more significant efficacy in suppressing the molecular changes observed in In Vitro and In Vivo models. In both cell lines, the drug combination showed synergistic effect at low doses. However, as the doses and exposure rates increased, the efficacy of the combination therapy decreased and became additive or antagonistic. These results show that the synergistic effect of the combination therapy is especially evident at low and medium doses.

These findings highlight the need for advanced clinical trials to assess the efficacy and safety of the combination therapy, including its long‐term effects and potential harm to healthy tissues. Future studies should focus on thoroughly investigating the long‐term toxicity and safety profile of this combination. Although TQ has demonstrated effectiveness even at low doses when combined with chemotherapy, its long‐term toxicity on healthy cells remains not fully determined. Additionally, since TQ is not currently available in oral or intravenous forms, research should aim to develop more stable and bioavailable formulations. Further investigation is also needed into the molecular mechanisms and targets of the TQ and 5‐FU combination, particularly how it affects cellular pathways in various cancer types and its contribution to disease progression. Finally, the potential effectiveness of this combination in cancers other than colorectal, such as breast, lung, or prostate cancer, should be explored.

## Author Contributions

**Eray Metin Guler:** conceptualization, investigation, writing – original draft, methodology, validation, visualization, writing – review and editing, data curation, project administration, formal analysis. **Kubra Bozali:** data curation, validation. **Onder Huseyinbas:** project administration. **Mert Celikten:** project administration. **Abdurrahim Kocyigit:** supervision, conceptualization, methodology.

## Ethics Statement

Ethics committee decision number 2017‐13 was taken from Bezmialem Vakif University Experimental Animal Ethics Committee.

## Conflicts of Interest

The authors declare no conflicts of interest.

## Data Availability

The data that support the findings of this study are available from the corresponding author upon reasonable request.

## References

[1] A. M. Noone , K. A. Cronin , S. F. Altekruse , et al., “Cancer Incidence and Survival Trends by Subtype Using Data From the Surveillance Epidemiology and End Results Program, 1992–2013,” Cancer Epidemiology, Biomarkers & Prevention 26, no. 4 (2017): 632–641. 10.1158/1055-9965.EPI-16-0520 PMC5380602 27956436

[2] R. F. C. Leitão , R. A. Ribeiro , E. A. L. Bellaguarda , et al., “Role of Nitric Oxide on Pathogenesis of 5‐Fluorouracil Induced Experimental Oral Mucositis in Hamster,” Cancer Chemotherapy and Pharmacology 59 (2007): 603–612. 16944152 10.1007/s00280-006-0301-y

[3] E. Rouhollahi , S. Z. Moghadamtousi , N. Al‐Henhena , et al., “The Chemopreventive Potential of Curcuma Purpurascens Rhizome in Reducing Azoxymethane‐Induced Aberrant Crypt Foci In Rats,” Drug Design, Development and Therapy no. 9 (2015): 3911–3922. 26251570 10.2147/DDDT.S84560 PMC4524378

[4] J. Ferlay , I. Soerjomataram , R. Dikshit , et al., “Cancer Incidence and Mortality Worldwide: Sources, Methods and Major Patterns in Globocan 2012,” International Journal of Cancer 136, no. 5 (2015): E359–E386. 25220842 10.1002/ijc.29210

[5] R. Labianca , B. Nordlinger , G. D. Beretta , A. Brouquet , and A. Cervantes , “Primary Colon Cancer: Esmo Clinical Practice Guidelines for Diagnosis, Adjuvant Treatment and Follow‐Up,” Annals of Oncology 21 (2010): v70–v77. 20555107 10.1093/annonc/mdq168

[6] G. Mitchell , S. Porter , and E. Manias , “From Telling to Sharing to Silence: A Longitudinal Ethnography of Professional‐Patient Communication about Oral Chemotherapy for Colorectal Cancer,” Psycho‐Oncology 28, no. 2 (2019): 336–342. 30444957 10.1002/pon.4945

[7] E. Van Cutsem , C. H. Köhne , E. Hitre , et al., “Cetuximab and Chemotherapy as Initial Treatment for Metastatic Colorectal Cancer,” New England Journal of Medicine 360, no. 14 (2009): 1408–1417. 19339720 10.1056/NEJMoa0805019

[8] D. J. Newman and G. M. Cragg , “Natural Products as Sources of New Drugs Over the 30 Years From 1981 to 2010,” Journal of Natural Products 75, no. 3 (2012): 311–335. 22316239 10.1021/np200906s PMC3721181

[9] M. J. Balunas and A. D. Kinghorn , “Drug Discovery From Medicinal Plants,” Life Sciences 78, no. 5 (2005): 431–441. 16198377 10.1016/j.lfs.2005.09.012

[10] C. P. Khare , Indian Herbal Remedies: Rational Western Therapy, Ayurvedic, and Other Traditional Usage, Botany (Springer Science & Business Media, 2004).

[11] B. H. Ali and G. Blunden , “Pharmacological and Toxicological Properties of *Nigella sativa* ,” Phytotherapy Research 17, no. 4 (2003): 299–305. 12722128 10.1002/ptr.1309

[12] M. S. Al‐Jassir , “Chemical Composition and Microflora of Black Cumin (*Nigella sativa* L.) Seeds Growing in Saudi Arabia,” Food Chemistry 45, no. 4 (1992): 239–242.

[13] A. Ahmad , A. Husain , M. Mujeeb , et al., “A Review on Therapeutic Potential of *Nigella sativa*: A Miracle Herb,” Asian Pacific Journal of Tropical Biomedicine 3, no. 5 (2013): 337–352. 23646296 10.1016/S2221-1691(13)60075-1 PMC3642442

[14] A. Kocyigit and E. M. Guler , “Curcumin Induce DNA Damage and Apoptosis Through Generation of Reactive Oxygen Species and Reducing Mitochondrial Membrane Potential in Melanoma Cancer Cells,” Cellular and Molecular Biology 63, no. 11 (2017): 97–105. 10.14715/cmb/2017.63.11.17 29208180

[15] H. Rottenberg and S. Wu , “Quantitative Assay by Flow Cytometry of the Mitochondrial Membrane Potential in Intact Cells,” Biochimica et Biophysica Acta—Molecular Cell Research 1404, no. 3 (1998): 393–404. 10.1016/s0167-4889(98)00088-3 9739168

[16] N. P. Singh , D. B. Danner , R. R. Tice , L. Brant , and E. L. Schneider , “DNA Damage and Repair With Age in Individual Human Lymphocytes,” Mutation Research/DNAging 237, no. 3–4 (1990): 123–130. 10.1016/0921-8734(90)90018-m 2233818

[17] E. M. Guler , B. H. Sisman , A. Kocyigit , and M. A. Hatiboglu , “Investigation of Cellular Effects of Thymoquinone on Glioma Cell,” Toxicology Reports 8 (2021): 162–170. 33489775 10.1016/j.toxrep.2020.12.026 PMC7806546

[18] L. D. C. Peiris , P. Chathu , D. D. B. D. Perera , and H. D. Moore , “1, 3‐Dinitrobenze‐Induced Genotoxicity Through Altering Nuclear Integrity of Diploid and Polyploidy Germ Cells,” Dose‐Response 17, no. 3 (2019): 155932581987676. 10.1177/1559325819876760 PMC6757507 31579111

[19] D. Peiris , I. Pacheco , C. Spencer , and R. J. MacLeod , “The Extracellular Calcium‐Sensing Receptor Reciprocally Regulates the Secretion of BMP‐2 and the Bmp Antagonist Noggin In Colonic Myofibroblasts,” American Journal of Physiology‐Gastrointestinal and Liver Physiology 292, no. 3 (2007): G753–G766. 17138967 10.1152/ajpgi.00225.2006

[20] O. Erel , “A New Automated Colorimetric Method for Measuring Total Oxidant Status,” Clinical Biochemistry 38, no. 12 (2005): 1103–1111. 16214125 10.1016/j.clinbiochem.2005.08.008

[21] O. Erel , “A Novel Automated Direct Measurement Method for Total Antioxidant Capacity Using a New Generation, More Stable ABTS Radical Cation,” Clinical Biochemistry 37, no. 4 (2004): 277–285. 15003729 10.1016/j.clinbiochem.2003.11.015

[22] O. Erel and S. Neselioglu , “A Novel and Automated Assay for Thiol/Disulphide Homeostasis,” Clinical Biochemistry 47, no. 18 (2014): 326–332. 25304913 10.1016/j.clinbiochem.2014.09.026

[23] M. A. Hatiboglu , A. Kocyigit , E. M. Guler , et al., “Thymoquinone Induces Apoptosis in B16‐F10 Melanoma Cell Through Inhibition of p‐STAT3 and Inhibits Tumor Growth in a Murine Intracerebral Melanoma Model,” World Neurosurgery 114 (2018): e182–e190. 29510292 10.1016/j.wneu.2018.02.136

[24] T. Wu , L. Qiang , F. H. Chen , et al., “LFG‐500, a Newly Synthesized Flavonoid, Induced a Reactive Oxygen Species‐Mitochondria‐Mediated Apoptosis in Hepatocarcinoma Cells,” Biomedicine & Preventive Nutrition 1, no. 2 (2011): 132–138.

[25] R. Agbaria , A. Gabarin , A. Dahan , and S. Ben‐Shabat , “Anticancer Activity of *Nigella sativa* (Black Seed) and Its Relationship With the Thermal Processing and Quinone Composition of the Seed,” Drug Design Development & Therapy 11, no. 9 (2015): 3119–3124. 10.2147/DDDT.S82938 PMC4476428 26124636

[26] S. Darakhshan , A. Bidmeshki Pour , A. Hosseinzadeh Colagar , and S. Sisakhtnezhad , “Thymoquinone and Its Therapeutic Potentials,” Pharmacological Research 95–96 (2015): 138–158. 10.1016/j.phrs.2015.03.011 25829334

[27] C. C. Woo , A. P. Kumar , G. Sethi , and K. H. B. Tan , “Thymoquinone: Potential Cure for Inflammatory Disorders and Cancer,” Biochemical Pharmacology 83, no. 4 (2012): 443–451. 22005518 10.1016/j.bcp.2011.09.029

[28] M. U. Nessa , P. Beale , C. Chan , J. Q. Yu , and F. Huq , “Synergism From Combinations of Cisplatin and Oxaliplatin With Quercetin and Thymoquinone in Human Ovarian Tumour Models,” Anticancer Research 31, no. 11 (2011): 3789–3797. 22110201

[29] S. H. Jafri , J. Glass , R. Shi , S. Zhang , M. Prince , and H. Kleiner‐Hancock , “Thymoquinone and Cisplatin as a Therapeutic Combination in Lung Cancer: In Vitro and In Vivo,” Journal of Experimental & Clinical Cancer Research 29, no. 1 (2010): 87. 20594324 10.1186/1756-9966-29-87 PMC2909169

[30] B. Ndreshkjana , A. Çapci , V. Klein , et al., “Combination of 5‐Fluorouracil and Thymoquinone Targets Stem Cell Gene Signature in Colorectal Cancer Cells,” Cell Death & Disease 10, no. 6 (2019): 379. 31097715 10.1038/s41419-019-1611-4 PMC6522523

[31] M. Acar , M. Gunduz , T. Ocak , et al., “Investigation of the Effects of 5‐Fluorouracil and Thymoquinone on Head and Neck Cancer Stem Cells,” Clinical and Investigative Medicine 38, no. 4 (2015): E209.

[32] X. Li , Z. Xun , and Y. Yang , “Inhibition of Phosphoserine Phosphatase Enhances the Anticancer Efficacy of 5‐Fluorouracil In Colorectal Cancer,” Biochemical and Biophysical Research Communications 477, no. 4 (2016): 633–639. 27349874 10.1016/j.bbrc.2016.06.112

[33] M. Wilhelm , L. Mueller , M. C. Miller , et al., “Prospective, Multicenter Study of 5‐Fluorouracil Therapeutic Drug Monitoring in Metastatic Colorectal Cancer Treated in Routine Clinical Practice,” Clinical Colorectal Cancer 15, no. 4 (2016): 381–388. 27256667 10.1016/j.clcc.2016.04.001

[34] N. El‐Najjar , M. Chatila , H. Moukadem , et al., “Reactive Oxygen Species Mediate Thymoquinone‐Induced Apoptosis and Activate ERK and JNK Signaling,” Apoptosis 15 (2010): 183–195. 19882352 10.1007/s10495-009-0421-z

[35] L. Salim , S. Mohan , R. Othman , et al., “Thymoquinone Induces Mitochondria‐Mediated Apoptosis in Acute Lymphoblastic Leukaemia In Vitro,” Molecules 18, no. 9 (2013): 11219–11240. 24036512 10.3390/molecules180911219 PMC6269888

[36] S. Ince , I. Kucukkurt , H. H. Demirel , R. Turkmen , and E. Sever , “Thymoquinone Attenuates Cypermethrin Induced Oxidative Stress in Swiss Albino Mice,” Pesticide Biochemistry and Physiology 104, no. 3 (2012): 229–235.

[37] J. M. Carethers , D. P. Chauhan , D. Fink , et al., “Mismatch Repair Proficiency and In Vitro Response to 5‐Fluorouracil,” Gastroenterology 117, no. 1 (1999): 123–131. 10381918 10.1016/s0016-5085(99)70558-5 PMC4343206

[38] M. Meyers , M. W. Wagner , A. Mazurek , C. Schmutte , R. Fishel , and D. A. Boothman , “Dna Mismatch Repair‐Dependent Response to Fluoropyrimidine‐Generated Damage,” Journal of Biological Chemistry 280, no. 7 (2005): 5516–5526. 15611052 10.1074/jbc.M412105200

[39] R. L. Gurung , S. N. Lim , A. K. Khaw , et al., “Thymoquinone Induces Telomere Shortening, DNA Damage and Apoptosis in Human Glioblastoma Cells,” PLoS One 5, no. 8 (2010): e12124. 20711342 10.1371/journal.pone.0012124 PMC2920825

[40] O. A. Kensara , A. G. El‐Shemi , A. M. Mohamed , B. Refaat , S. Idris , and J. Ahmad , “Thymoquinone Subdues Tumor Growth and Potentiates the Chemopreventive Effect of 5‐Fluorouracil on the Early Stages of Colorectal Carcinogenesis in Rats,” Drug Design Developmen & Therapy 11, no. 10 (July 2016): 2239–2253. 10.2147/DDDT.S109721 PMC4946859 27468227

[41] S. P. Trachana , E. Pilalis , N. G. Gavalas , et al., “The Development of An Angiogenic Protein 'Signature' in Ovarian Cancer Ascites as a Tool for Biologic and Prognostic Profiling,” PLoS One 11, no. 6 (2016): e0156403. 27258020 10.1371/journal.pone.0156403 PMC4892506

[42] A. Parveen , L. Subedi , H. W. Kim , et al., “Phytochemicals Targeting VEGF and VEGF‐Related Multifactors as Anticancer Therapy,” Journal of Clinical Medicine 8, no. 3 (2019): 350. 30871059 10.3390/jcm8030350 PMC6462934

[43] V. Cecarini , L. Quassinti , A. Di Blasio , et al., “Effects of Thymoquinone on Isolated and Cellular Proteasomes,” FEBS journal 277, no. 9 (2010): 2128–2141. 20412058 10.1111/j.1742-4658.2010.07629.x

[44] E. M. Dergarabetian , K. I. Ghattass , S. B. El‐Sitt , et al., “Thymoquinone Induces Apoptosis in Malignant T‐Cells via Generation of ROS,” Frontiers in Bioscience 5, no. 2 (2013): 706–719. 10.2741/e651 23277025

[45] C. Buhrmann , M. Yazdi , B. Popper , et al., “Resveratrol Chemosensitizes TNF‐β‐induced Survival of 5‐FU‐treated Colorectal Cancer Cells,” Nutrients 10, no. 7 (2018): 888. 30002278 10.3390/nu10070888 PMC6073304

[46] H. Zhang , J. Tang , C. Li , et al., “MiR‐22 Regulates 5‐FU Sensitivity by Inhibiting Autophagy and Promoting Apoptosis in Colorectal Cancer Cells,” Cancer Letters 356, no. 2 (2015): 781–790. 25449431 10.1016/j.canlet.2014.10.029

[47] A. Al Shoyaib , S. R. Archie , and V. T. Karamyan , “Intraperitoneal Route of Drug Administration: Should It Be Used in Experimental Animal Studies?,” Pharmaceutical Research 37, no. 1 (2020): 12. 10.1007/s11095-019-2745-x PMC7412579 31873819

